# 2-Hydroxy-4-methoxybenzaldehyde, a more effective antifungal aroma than vanillin and its derivatives against *Fusarium graminearum*, destroys cell membranes, inhibits DON biosynthesis, and performs a promising antifungal effect on wheat grains

**DOI:** 10.3389/fmicb.2024.1359947

**Published:** 2024-02-26

**Authors:** Qian Li, Chong Wang, Hongying Xiao, Yiming Zhang, Yanli Xie

**Affiliations:** ^1^Grain, Oil and Food Engineering Technology Research Center of the State Grain and Reserves Administration/Key Laboratory of Henan Province, Henan University of Technology, Zhengzhou, Henan, China; ^2^Henan Key laboratory of Cereal and Oil Food Safety and Nutrition, College of Food Science and Engineering, Henan University of Technology, Zhengzhou, Henan, China

**Keywords:** 2-hydroxy-4-methoxybenzaldehyde, antifungal mechanism, *Fusarium graminearum*, wheat grain, vanillin and its derivatives

## Abstract

*Fusarium graminearum* (*F. graminearum*) is a severe pathogen threatening the safety of agriculture and food. This study aimed to explore the antifungal efficacies of several plant-derived natural compounds (vanillin and its derivatives) against the growth of *F. graminearum* and investigate the antifungal mechanism of 2-hydroxy-4-methoxybenzaldehyde (HMB), the strongest one. The minimum inhibitory concentration (MIC) of HMB in inhibiting mycelial growth was 200 μg/mL. HMB at MIC damaged cell membranes by increasing the permeability by about 6-fold (*p* < 0.05) as evidenced by propidium iodide (PI) staining. Meanwhile, the content of malondialdehyde (MDA) and glycerol was increased by 45.91 and 576.19% by HMB treatment at MIC, respectively, indicating that lipid oxidation and osmotic stress occurred in the cell membrane. Furthermore, HMB exerted a strong antitoxigenic role as the content of deoxynivalenol (DON) was remarkably reduced by 93.59% at MIC on 7th day. At last, the antifungal effect of HMB against *F. graminearum* was also confirmed on wheat grains. These results not only revealed the antifungal mechanism of HMB but also suggested that HMB could be applied as a promising antifungal agent in the preservation of agricultural products.

## Graphical abstract

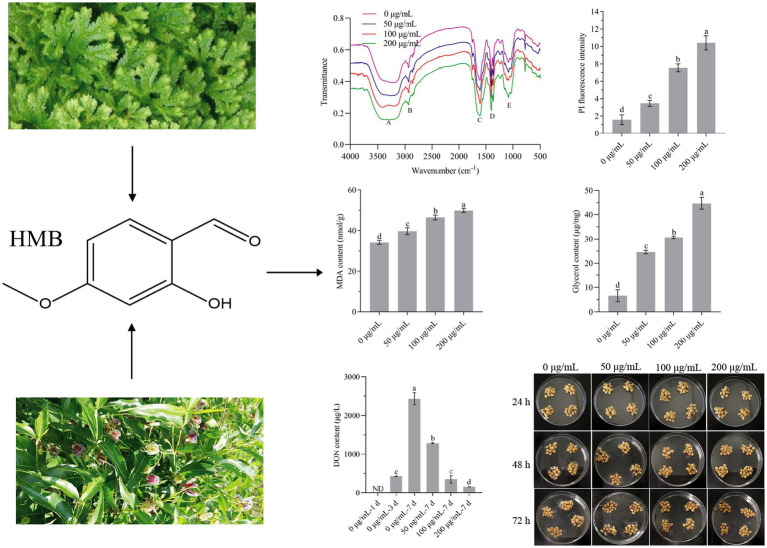

## Introduction

*Fusarium graminearum* (*F. graminearum*) belongs to deuteromycotina (imperfect fungi) in *Fusarium* spp., and is a common filamentous pathogen that causes Fusarium head blight (FHB; Chen et al., 2022; Tian et al., 2022). FHB is a devastating disease threatening wheat and other small cereal grains and therefore causes huge yield reduction and economic losses worldwide. In the USA, from the beginning of the 90’s of the 20th century to 2008, the wheat yield loss caused by FHB was estimated to reach US $3 billion (Windels, 2000). Since 2000, the occurrence of FHB has increased in China, and till 2018, 4.5 million hectares were affected, accounting for approximately 20% of the total planted area of wheat, and the yield loss per year exceeded 3.41 million tons (Chen et al., 2019).

Apart from the strain itself, various mycotoxins are produced through its secondary metabolism, mainly including trichothecenes and zearalenones, which are toxic to humans and livestock (Chen et al., 2022). Ingestion of these mycotoxin-contaminated unprocessed and processed grains usually causes detrimental health effects, such as immunosuppression, infertility, nephropathy, cancer, or even death (Wu et al., 2017). As these Fusarium mycotoxins are thermally stable, common food processing methods are not able to degrade and/or remove them. At present, among various strategies for controlling FHB, chemical fungicides are the primary means, such as carbendazim, tebuconazole, flutriafol, etc. (Moonjely et al., 2023). However, resistant fungi strains have been detected in a growing trend in the past 10 years in China, and such fungicides are not friendly to environmental safety and human health (Sun et al., 2018; Zhang et al., 2020). Therefore, it is an urgent prerequisite to search for safe methods.

**Figure 1:**
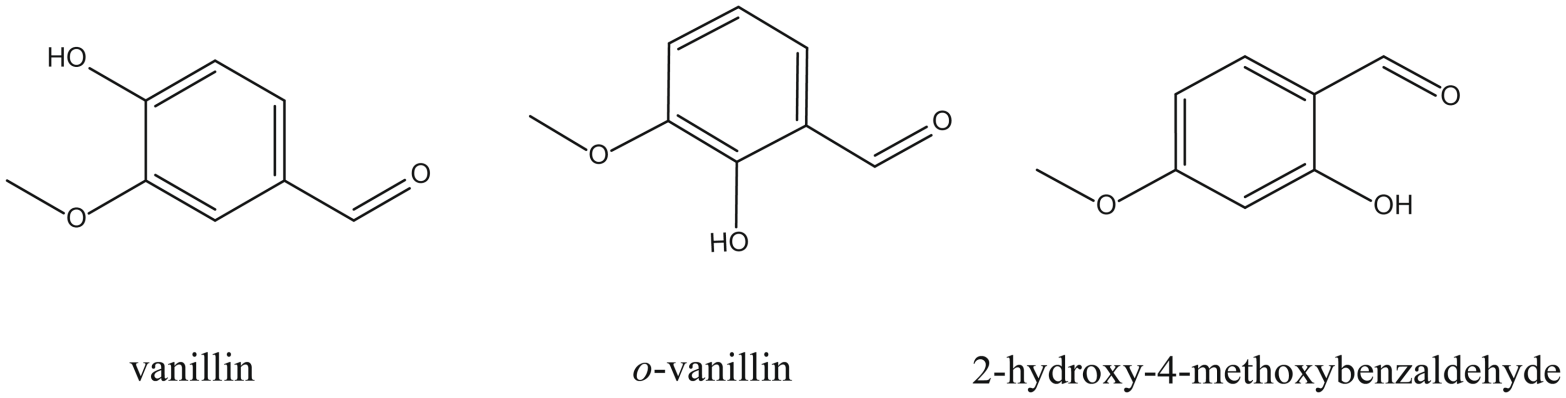
Chemical structures of vanillin, *o*-vanillin, and HMB.

Plant-derived compounds are superior to chemical fungicides due to many advantages, such as high-yield, effective, eco-friendly, etc. (Arunachalam et al., 2023). Representative compounds, such as thymol (Gao et al., 2016), ferulic acid (Yan et al., 2023), and camphor (Kong et al., 2022) have been reported to inhibit the growth of *F. graminearum* by disrupting the integrity of cell membranes. Vanillin, *o*-vanillin and 2-hydroxy-4-methoxybenzaldehyde (HMB) are isomers (C_8_H_8_O_3_) derived from benzaldehydes (chemical structures shown in Figure 1) and have broad antimicrobial spectrum, including *Aspergillus* spp. (López-Malo et al., 1997; Kim et al., 2011), *Penicillium* spp. (Scora and Scora, 1998; Matamoros-León et al., 1999; Mohana and Raveesha, 2010), and *Cryptococcus neoformans* (Kim et al., 2014). Interestingly, many recent studies suggested that cell membrane is a potential antifungal target of vanillin and its derivatives in various conditional pathogens [e.g., *Botrytis cinerea* and *Alternaria alternata* (Yang J. et al., 2021)], *Escherichia coli* (*E. coli*) O157:H7 (Chen et al., 2023), *Aspergillus flavus* (*A. flavus*; Li et al., 2021d), and *Staphylococcus aureus* (Kannappan et al., 2023). Our previous study demonstrated that vanillin and its derivatives performed strong antifungal effects on *A. flavus* and among them, HMB was the most effective one (Li and Zhu, 2021). HMB is a flavor compound found in the roots and rhizomes of many medicinal plants, such as *Decalipus hamiltonii*, *Hemidesmus indicus*, *Mondia whitei*, *Periploca sepium*, and *Sclerocarya caffra*, and it has been reported to play roles in many biological functions, such as antimicrobial, anti-inflammatory, hepatoprotective, neuroprotective, etc. (Rathi et al., 2017). In addition, HMB is a generally regarded as safe (GRAS) reagent and applied as a flavoring agent, adjuvant or medicine (FDA, 2021). Consequently, it is supposed to be a promising antifungal agent applied in food processing and preservation. A recent study reported that the antifungal effect of HMB against *A. flavus* was attributed to the damage of the integrities of cell walls and cell membranes as well as suppression of respiration (Li et al., 2021c). Although the antiaflatoxigenic activity of HMB on *A. flavus* has also been suggested (Li et al., 2021c), its mode of action on *F. graminearum* is still unknown. To screen out the strongest aroma among vanillin and its derivatives and explore the potential antifungal target, in this study, the antifungal efficacy of vanillin, *o*-vanillin, and HMB against *F. graminearum* was first compared, then the effects of HMB on the cell surface and the integrity of cell membranes were determined. Finally, the content of deoxynivalenol (DON) was quantified and the antifungal effect on wheat grains against *F. graminearum* was evaluated.

## Materials and methods

### Fungal species

*Fusarium graminearum* PH-1 was kindly gifted from the food microbiological hazard control lab at Henan University of Technology. The strain was cultured with Potato Dextrose Agar (PDA) containing 200 g/L of potato, 20 g/L dextrose, and 15 g/L of agar at 28°C ± 2°C. Mycelia used for experiments in this study were cultured with Potato Dextrose Broth (PDB) containing 200 g/L of potato and 20 g/L dextrose. Spores were collected as follows: 5 mycelial plugs (6 mm diameter) were inoculated into CMC (carboxymethylcellulose sodium medium) containing 15 g/L of carboxymethylcellulose sodium, 1 g/L of yeast extract, 1 g/L of NH_4_NO_3_, 1 g/L of KH_2_PO_4_, and 0.5 g/L of MgSO_4_•7H_2_O. After shaking cultivated at 180 r/min for 7 days, the filtrate was collected by 3 layers of lens wiping paper. Afterwards, the filtrate was centrifuged at 3,000 r/min for 10 min, the precipitation was collected and the concentration of spores was adjusted with a hemocytometer to the desired numbers.

### Chemicals

Vanillin (98%, CAS: 121-33-5), *o*-vanillin (99%, CAS: 148-53-8), HMB (98%, CAS: 673-22-3), and propidium iodide (PI) were purchased from Aladdin BioChem Technology Co. (Shanghai, China).

### Susceptibility test

Effects of vanillin, *o*-vanillin, and HMB on mycelial growth of *F. graminearum* were determined *in vitro* as described previously (Gao et al., 2016). In brief, the above three aromas were mixed with PDA (20 mL) and poured into sterilized Petri dishes (80 mm diameter). The final concentrations of vanillin were 0, 200, 400, 800, 1,200, and 1,600 μg/mL, the final concentrations of *o*-vanillin were 0, 50, 100, 200, 300, and 400 μg/mL, the final concentrations of HMB were 0, 40, 80, 120, 160, and 200 μg/mL. A mycelial plug (6 mm diameter) from a one-week-old culture plate was cut off and inoculated into the center of an aroma-PDA plate. The mycelial growth inhibition rate was calculated as the following formula:


$$\mathrm{MGI}\;\left(\%\right)=\frac{\left(d_{c}-0.6\right)-\left(d_{t}-0.6\right)}{d_{c}-0.6}\times 100$$

Where d_c_ (cm) is the average colony diameter in the control group (without aroma addition), and d_t_ is the average colony diameter in the treatment groups. Minimum inhibitory concentration (MIC) was considered as the MGI was 100% after 72 h.

The dry weight of mycelia was determined as follows: 1 mL of spore suspension (10^5^ spores/mL) was inoculated in 100 mL of PDB, after shaking cultivated at 150 r/min for 24 h, HMB was added to reach the final concentrations of 0, 50, 100, and 200 μg/mL. After another cultivation for 24 h, mycelia were collected, dried (80°C) and weighed.

### Fourier transform infrared spectroscopy

Fourier transform infrared spectroscopy (FT-IR) characterization was conducted as described previously (Li et al., 2021d). In brief, 1 mg of mycelial lyophilized powder was mixed with 100 mg of KBr and pressed into a sheet. An FT-IR spectrophotometer (Alpha, Bruker Corp., Karlsruhe, Germany) was used to collect the spectra.

### Cell membrane integrity test

PI staining was conducted as the protocol provided by the supplier. Fresh mycelia were collected and stained with PI (1 μL/mL) for 20 min in darkness. Samples were ready for visualization with a fluorescence microscope (DFC 7000 T, Leica, Germany) after extensive washing.

### Determination of relative conductivity and pH value

The relative conductivity and pH value were investigated as described previously with slight modifications (Li et al., 2021b). Briefly, fresh mycelia were collected and washed twice with pure water. Five hundred micrograms of mycelia were suspended in 20 mL pure water and the conductivity was measured at 0, 30, 60, 90, 120, 150, and 180 min with a conductivity meter (DZS-706-C, INESA Scientific Instrument Co., Ltd., Shanghai, China). Finally, the conductivity of mycelia after boiling for 5 min was also measured. The relative conductivity was calculated as the following formula:


$$\mathrm{Relative conductivity}\left(\%\right)=\frac{C_{1}-C_{a}}{C_{2}-C_{b}}\times 100$$

Where C_1_ and C_2_ represent the conductivities of mycelia before and after boiling, respectively. C_a_ and C_b_ represent the conductivities of pure water before and after boiling, respectively.

The pH values were determined right after conductivity.

### Determination of the content of malondialdehyde and glycerol

The content of malondialdehyde (MDA) was measured with a malondialdehyde content assay kit (BC0020, Solarbio Science & Technology Co. Beijing, China). The content of glycerol was measured as the method described previously (Lynch and Yang, 2004). In brief, 10 mg of lyophilized mycelia was dissolved in 4 mL of petroleum ether and 4 mL of 50% ethanol. This procedure was followed by vortexing for 5 min and centrifugation at 3,000 r/min for 10 min, 100 μL of the lower layer was mixed with 900 μL of 50% ethanol. Then, 1 mL of 0.015 mol/L KIO_4_ was added, and after incubation for 10 min, 2 mL of 5.5 mM/L L-rhamnose monohydrate and 4 mL of Nash reagent (150 g of ammonium acetate, 2 mL of acetic acid, and 2 mL of acetylacetone dissolved in distilled water to 1 L) was added. Lastly, the mixture was incubated in a water bath at 53°C for 15 min, and the absorbance of samples was measured with a UV/Vis spectrophotometer (UV-6100S, Mapada, Shanghai, China).

### Determination of DON content

The content of DON was determined as the method described previously (Song et al., 2014). Spores were added in 100 mL of PDB to reach the final concentration of 10^5^ spores/mL. After shaking cultivation for 24 h, HMB was added to reach the final concentrations of 0, 50, 100, and 200 μg/mL. After 2, 4, 6 days of cultivation, the culture medium was collected by centrifugation at 8000 r/min and DON was extracted by using the DON immunoaffinity column according to the manuscript provided by the supplier (GYIC-030-3, guanyibio, Wuxi, China). The content of DON was determined by High-performance Liquid Chromatography (HPLC, Agilent 1,260 Infinity II, Agilent Technologies, China) with Eclipse Plus C18 column (250 mm × 4.6 mm, 5 μm) according to GB 5009.111-2016. The detection conditions were as follows: column temperature 35°C, mobile phase water and methanol mixture (v:v = 7:3), flow rate 1 mL/min, detection wavelength 218 nm.

### Antifungal effect of HMB on wheat grains *in vitro*

Wheat grains were sterilized with 0.5% sodium hypochlorite and washed extensively with sterilized water, then, UV light was introduced to irradiate the wheat grains for 20 min. Afterward, 60 wheat grains were mixed with spore suspension (10^5^ spores/mL) and HMB (total volume: 1.2 mL), placed in Petri dishes (diameter: 9 cm), and cultivated for 72 h. The germination of spores on wheat grains was observed every 24 h. Finally, 0.9% NaCl was used to wash the wheat grains and the survival spores were collected and diluted, the viability of spores was calculated after cultivation for 24 h.

### Statistical analysis

The results are presented as the mean ± SD. The statistical analyses were performed using SPSS 20.0 (IBM, Armonk, United States), and the significant differences between mean values were calculated by one-way ANOVA using Duncan’s multiple range test. Pearson’s correlation analysis was used to evaluate the correlation between the content of MDA and DON.

## Results and discussion

### HMB exerted a stronger antifungal effect against *Fusarium graminearum* than vanillin and *o*-vanillin

**Figure 2:**
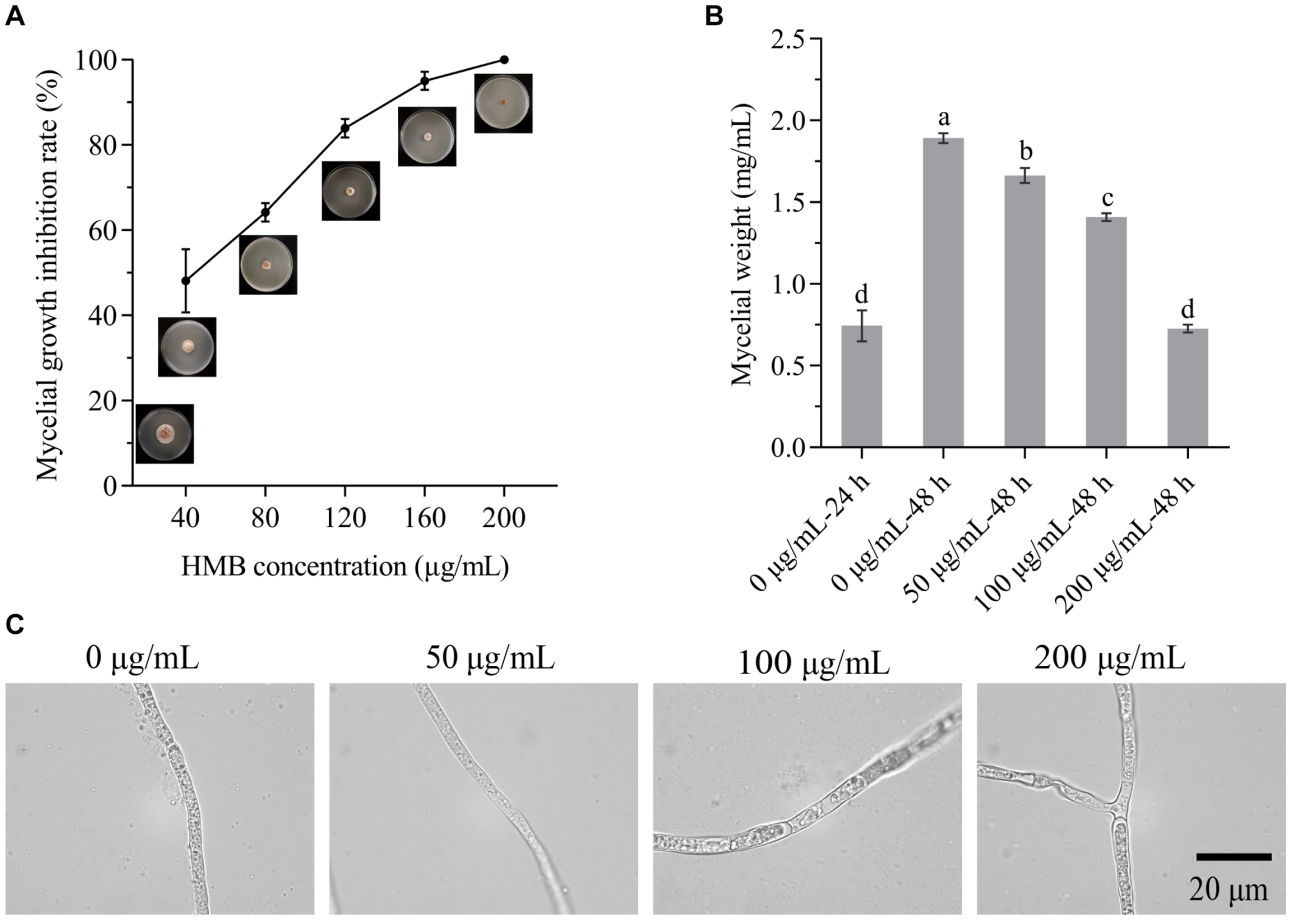
Inhibitory effect of HMB on *Fusarium graminearum*. **(A)** Mycelial growth inhibition rate. **(B)** Mycelial weight. **(C)** Mycelial morphology under optical microscope. Scale bar: 20 μm. ^a–d^Significant difference (*p* < 0.05) according to Duncan’s multiple range test.

To evaluate the antifungal strength of vanillin and its derivatives against the growth of *F. graminearum*, the MIC was first determined in a time-course experiment. As shown in Supplementary Table S1, the growth of *F. graminearum* was normal when no aroma was added to the PDA plate, while it was completely inhibited by vanillin, *o*-vanillin, and HMB at the concentrations of 1,600, 400, and 200 μg/mL within 72 h, hence, HMB exerted the greatest antifungal effect and its MIC was 200 μg/mL. This phenomenon was similar to that on *A. flavus*, which might be attributed to the relative positions of hydroxyl, aldehyde, and methoxy group on the benzene ring (Li and Zhu, 2021). Figure 2A shows the growth of inoculated mycelial plug treated with different concentrations of HMB at 72 h. The MIC of HMB was much higher than those of commercial fungicides, such as carbendazim, metconazole, and prochloraz (10 μg/mL; Ivić et al., 2011). Meanwhile, the MIC of HMB was higher than that of thymol (Gao et al., 2016) and ferulic acid (Yan et al., 2023; about 100 μg/mL) at 72 h against *F. graminearum*, but much lower than that of camphor (Kong et al., 2022; 4 mg/mL) at 8 days. The difference is probably due to the use of different species, inoculation concentration, and temperature. Still, strategies for reducing the dosage of HMB are of great interest. As expected, the mycelia weight (0.73 ± 0.02 mg/mL) when treated with HMB at 200 μg/mL for 24 h was similar (*p* > 0.05) as that of the control (0.77 ± 0.09 mg/mL) cultured for 24 h (Figure 2B), the treatment method was used for most of the following experiments. The morphology of mycelia treated with HMB was further examined with optical visualization (Figure 2C). Slight difference was found between the control and the 50 μg/mL group. However, obvious chaos of cytoplasm was observed in 100 and 200 μg/mL groups and large vacuoles were present in the mycelia, indicating the injury of HMB at high concentrations on the inner components/structures of mycelia.

### HMB damaged the morphology of mycelia

**Figure 3:**
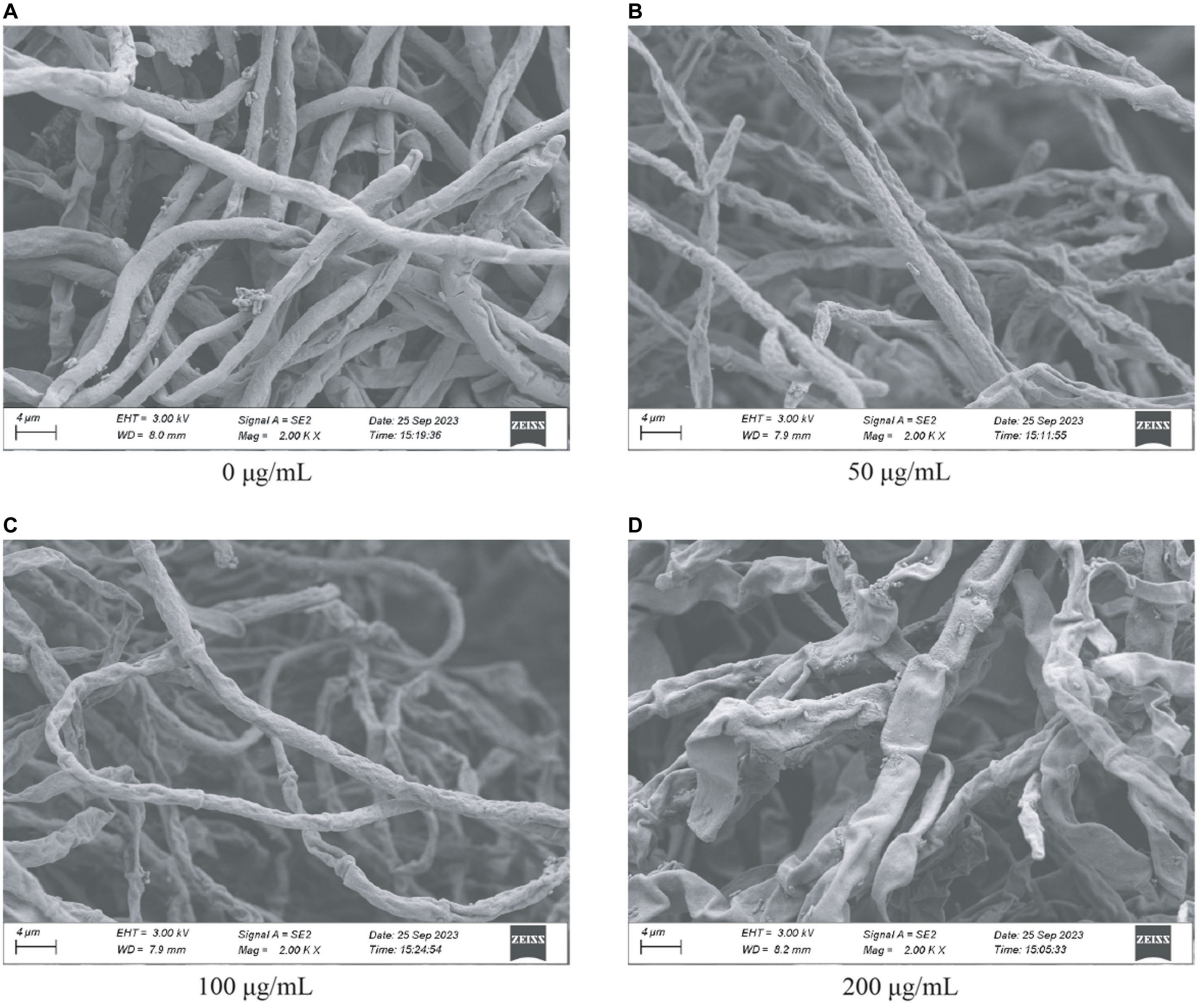
SEM characterization of *Fusarium graminearum* treated with HMB at **(A)** 0 μg/mL, **(B)** 50 μg/mL, **(C)** 100 μg/mL, and **(D)** 200 μg/mL.

To better evaluate the role of HMB in inhibiting the mycelia growth of *F. graminearum*, scanning electron microscopy (SEM) was adopted to examine the change of ultrastructure of the mycelia. As in Figure 3A, mycelia in the control group appeared strong and the surface was smooth. Shrunken mycelia with irregular structures were observed in the 50 μg/mL (Figure 3B) and 100 μg/mL (Figure 3C) groups. When the concentration of HMB increased to 200 μg/mL, a more remarkable damage effect was observed on mycelia, resulting in a flattened appearance with severe deformation and distortion (Figure 3D). Therefore, the injury effect of HMB on *F. graminearum* mycelia was confirmed, especially in the high concentration groups. Several researchers have stated that such loss of integrity and linearity of mycelia was usually attributed to the inhibition of enzymes in cell wall synthesis and/or leakage of intracellular constituents (Yahyazadeh et al., 2007; Sun et al., 2016; Lakshmeesha et al., 2019; Wan et al., 2019).

### HMB did not alter the surface functional groups of *Fusarium graminearum* mycelia

**Figure 4:**
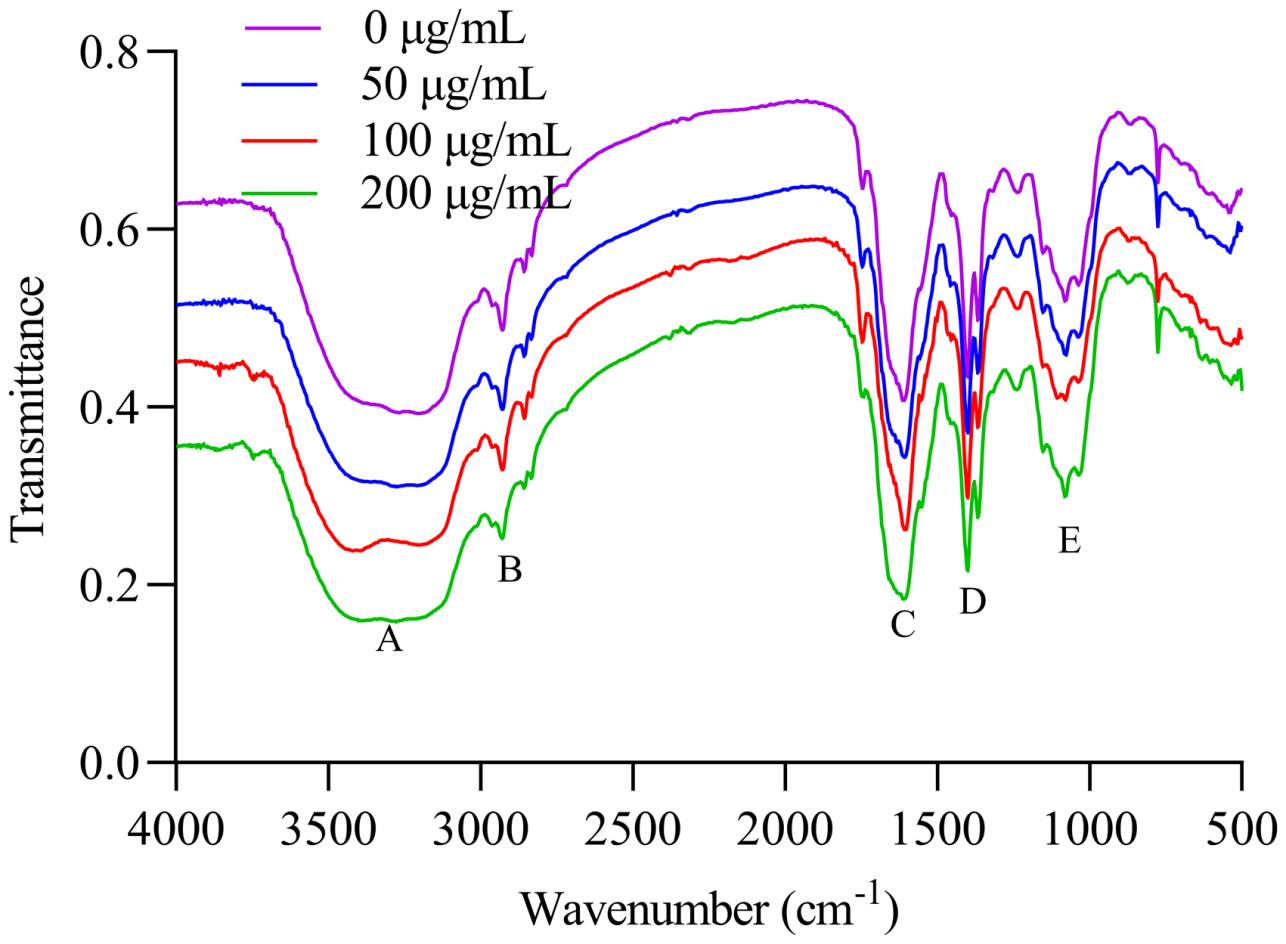
FT-IR spectra of *Fusarium graminearum* mycelia treated with HMB.

The amorphous layer on the outer surfaces of cell walls (also called fibrous decorations) was observed in *A. flavus* (Miyazawa et al., 2019; Li et al., 2021d), which was also shown in PH-1 strain (Wang et al., 2019). Although the components have not been clarified, the functional groups could be determined by FT-IR characterization and the change of which might direct further analysis of major components (Li et al., 2021a,d). To evaluate whether HMB changed the surface functional groups of mycelia of *F. graminearum*, FT-IR was conducted. As shown in Figure 4A, the broad absorption band in the range of between 3,450 and 3,170 cm^−1^ for the control and HMB-treated groups represented hydrogen bonds (Nandiyanto et al., 2019). The signal for the stretching vibration of CH_2_ occurs at 2,927 cm^−1^ (Li et al., 2019), as shown in Figure 4B. A strong absorption peak at 1,601 cm^−1^ (Figure 4C) and a following weak absorption peak at 1,550 cm^−1^ corresponded to the C=O stretching (amide I band) and -NH bending vibration (amide II band). The absorption peaks at 1,399 cm^−1^ (Figure 4D) and 1,076 cm^−1^ (Figure 4E) corresponded to trimethyl groups and C-C in the glucose chain. No significant movement of the wavenumbers was observed among the control and all HMB-treated groups; hence, it is suggested that the surface functional groups were not altered by HMB treatment, which was different with either *o*-vanillin or paeonol-treated *A. flavus* (Li et al., 2021a,d). The morphology of the amorphous layer of *F. graminearum* mycelia and whether it is changed by HMB treatment require further demonstration (e.g., TEM and component analysis).

### HMB disrupted the integrity of cell membranes, changed extracellular relative conductivity and pH value

**Figure 5:**
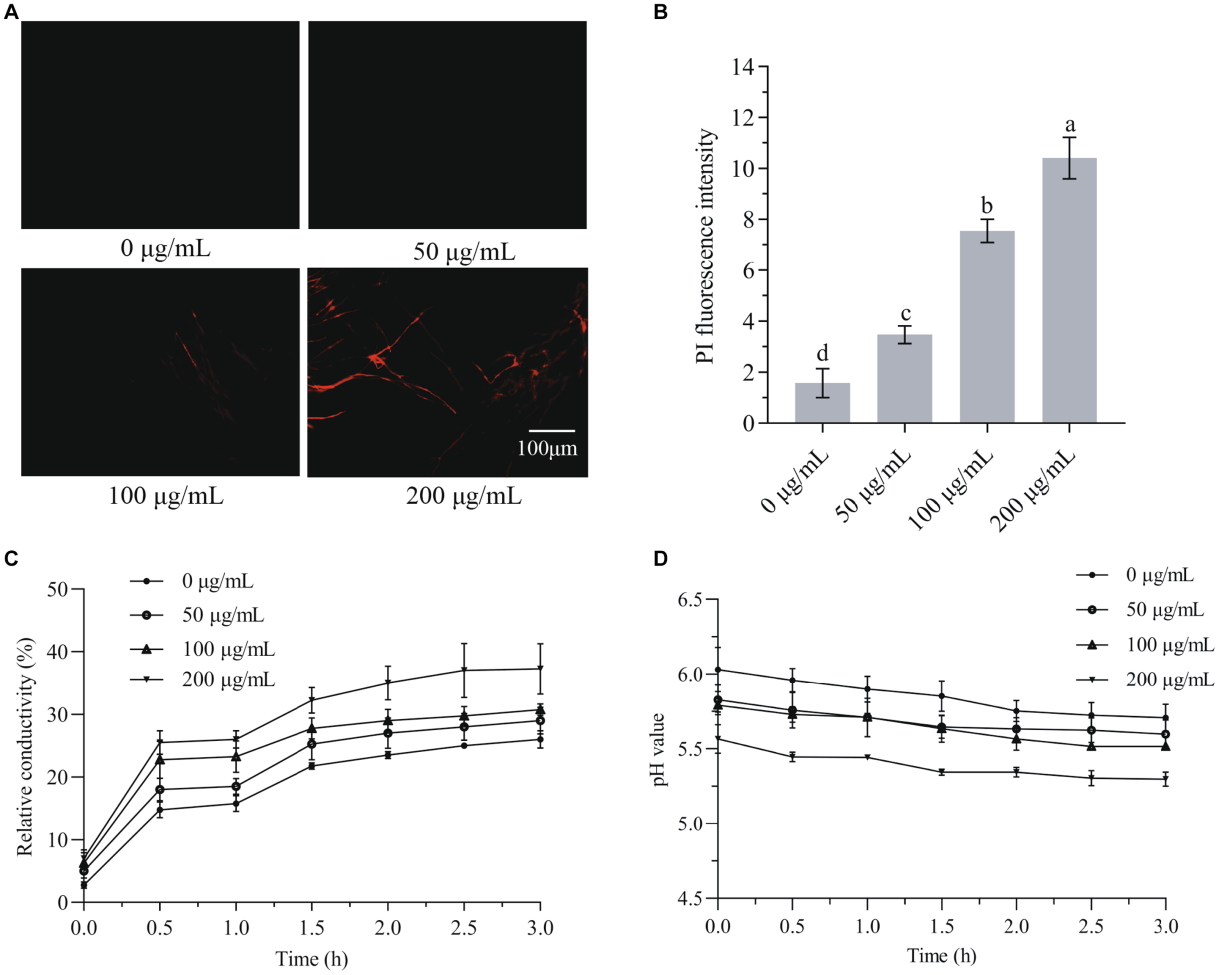
Effect of HMB on cell membrane integrity of *Fusarium graminearum*. **(A)** PI staining. **(B)** PI fluorescence intensity. **(C)** The relative conductivity value. **(D)** The extracellular pH. a-d significant difference (*p* < 0.05) according to Duncan’s multiple range test.

The hydrophobicity property of plant-derived aldehydes enables their binding with cell membranes and hence induces a higher membrane permeability (Burt, 2004). As HMB is hydrophobic in nature, it is assumed to affect the integrity of mycelial cell membranes. Hence, PI staining was first conducted and the fluorescence intensity was quantified. As shown in Figure 5A, fluorescence was observed when mycelia treated with HMB not lower than 100 μg/mL. The fluorescence intensity increased significantly (*p* < 0.05) with the increasing concentration of HMB, at 50, 100, and 200 μg/mL, the intensities were about 2, 5, and 7-fold of the control (Figure 5B). This observation suggests a direct injury of HMB on cell membranes. Furthermore, this finding led to the hypothesis that cell constituents (e.g., proteins and nucleic acid) were released out of mycelia. As in Figure 5C, the relative conductivities of the HMB-treated groups during the test time were always higher than the control group. Moreover, HMB treatment showed a dose-dependent manner. In detail, after incubation for 180 min, the relative conductivities of 100 and 200 μg/mL groups were 29.95 ± 0.73 and 37.34% ± 4.24%, respectively, which were much higher than that of the control (26.09% ± 1.17%). A similar growing trend of relative conductivity in *F. graminearum* was also found in many natural and synthetic antifungal agents, such as glabridin (Yang J. et al., 2021), guaiacol (Gao et al., 2021), ferulic acid (Yan et al., 2023), a quinoline derivative (Yang Y. D. et al., 2021), and ethylenediaminetetraacetic acid disodium salt (EDTANa_2_; Song et al., 2020). The pH value was determined to evaluate the acid–base property of the cellular leakage, and the results are shown in Figure 5D. The overall values of HMB-treated groups were lower than that of the control, where the 200 μg/mL group ranked as the lowest one, indicating that more acidic components were released out of mycelia after HMB treatment. This result was similar to that of HMB-treated *A. flavus* (Li et al., 2021c), although different antifungal agents and fungal species presented differently (Tao et al., 2014; Zhou et al., 2014).

### HMB induced nucleic acid and protein leakage

The determination of the optical density (OD) value could reflect the optical concentration of a solution, such as the nucleic acid and protein at the wavelengths of 260 and 280 nm. As the integrity of the cell membrane of *F. graminearum* was injured by HMB, the OD_260_ and OD_280_ of the culture medium were determined. As in Table 1, within the first 24 h, the OD_260_ and OD_280_ showed a similar trend. In detail, the OD_260_ value of the control group was 0.26 ± 0.01, no significant differences were found among the control group and the 50 and 100 μg/mL HMB treatment groups. However, HMB at 200 μg/mL significantly increased the OD_260_ value up to 0.36 ± 0.03 in comparison with either the control group (about 38.5% increase) or other HMB treatment groups. For OD_280_, about a 21.9% increase was determined for the 200 μg/mL group in comparison with the control group. The above results indicated that HMB at a high concentration (not lower than 200 μg/mL) promoted membrane permeability, resulting in a great loss of both nucleic acid and protein. These findings are consistent with previous studies on several plant extracts that they might also induce lipid oxidation and osmotic imbalance of cell membrane (Qu et al., 2019; Ju et al., 2020a).

**Table 1:** Effect of HMB on the cell permeability of *Fusarium graminearum*.

Concentration (μg/mL)	OD_260_	OD_280_
0	0.26 ± 0.01 b	0.32 ± 0.01 b
50	0.26 ± 0.00 b	0.34 ± 0.02 b
100	0.28 ± 0.01 b	0.35 ± 0.00 b
200	0.36 ± 0.03 a	0.39 ± 0.00 a

a-d significant difference (*p* < 0.05) according to Duncan’s multiple range test.

### HMB induced lipid oxidation and osmotic stress

**Figure 6:**
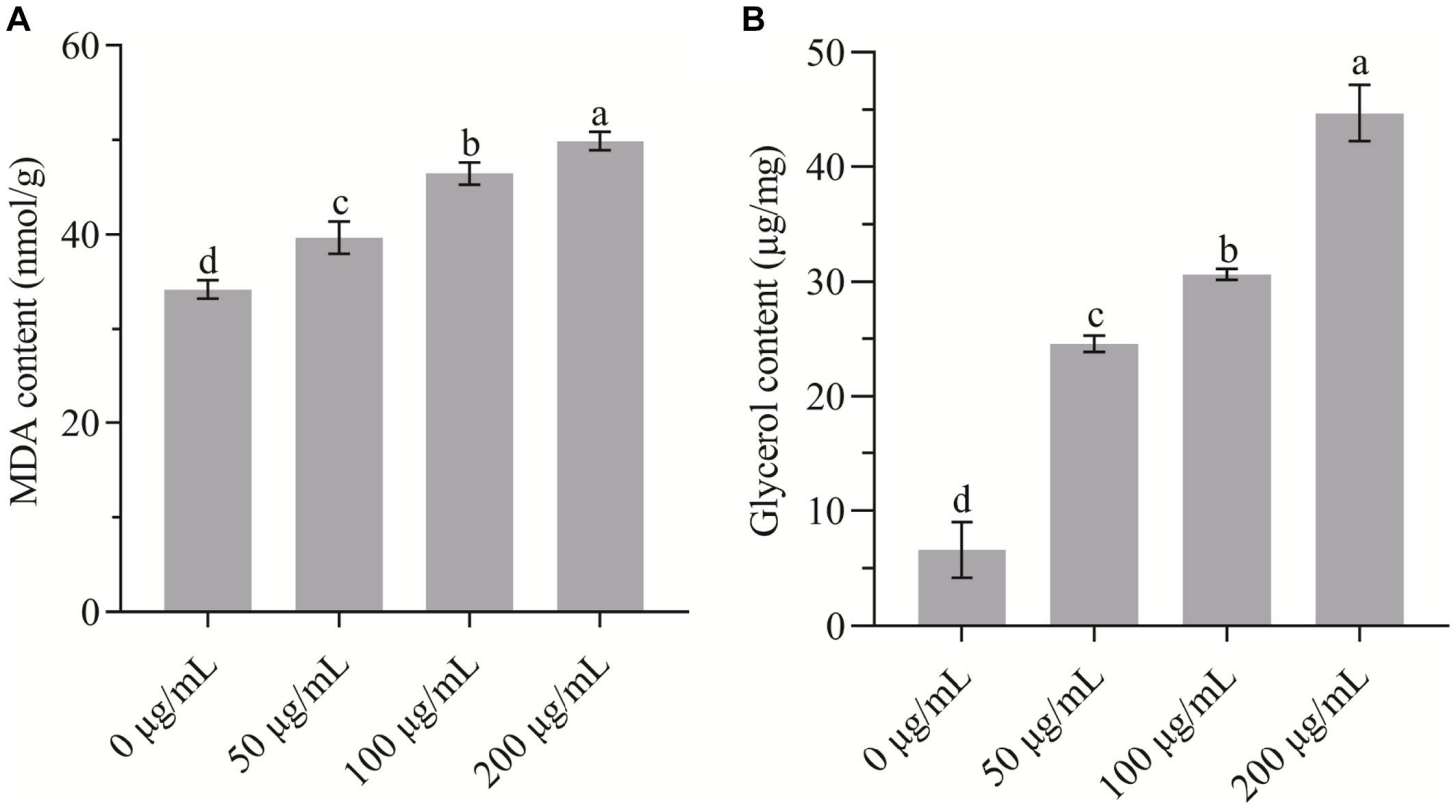
Determination of the content of **(A)** MDA and **(B)** glycerol of *Fusarium graminearum* treated with HMB. a-d significant difference (*p* < 0.05) according to Duncan’s multiple range test.

The content of MDA usually reflects the extent of lipid peroxidation that underlies the oxidative injury of the cell membrane. In the present study, as shown in Figure 6A, the MDA concentration was increased remarkably (*p* < 0.05) in a dose-dependent manner with HMB treatment. In comparison with the control group (34.19 ± 0.97 mmol/g), the values of the 50, 100, and 200 μg/mL groups increased by 16.04 ± 4.99, 35.85 ± 3.40, and 45.91% ± 2.89%, respectively. Although the increase of MDA content was found in many natural product treated pathogens (Ju et al., 2020b; Yin et al., 2021; Niu et al., 2022; Bao et al., 2023), Gao et al. reported that guaiacol exhibited an opposite pattern as it served as an antioxidant (Gao et al., 2021). To examine the osmotic stress response across cell membranes induced by HMB, the change of glycerol content was quantified. As shown in Figure 6B, in comparison with the control group (6.61 ± 1.41 μg/mL), the content of the 50, 100, and 200 μg/mL groups increased by about 3, 4, and 6 times, respectively. Collectively, we assume that the binding of HMB with cell membranes increased membrane permeability and induced a leakage of electrolytes, resulting in lipid oxidation and osmotic stress responses. Kim et al. depicted that as a redox-active molecule, HMB interfered cellular redox homeostasis and the HOG-MAPK signaling pathway (Kim et al., 2020). Therefore, a comprehensive elucidation of HMB in modulating the abovementioned signaling pathway (e.g., oxidation–reduction and osmotic stresses) is required.

### HMB decreased the content of DON

**Figure 7:**
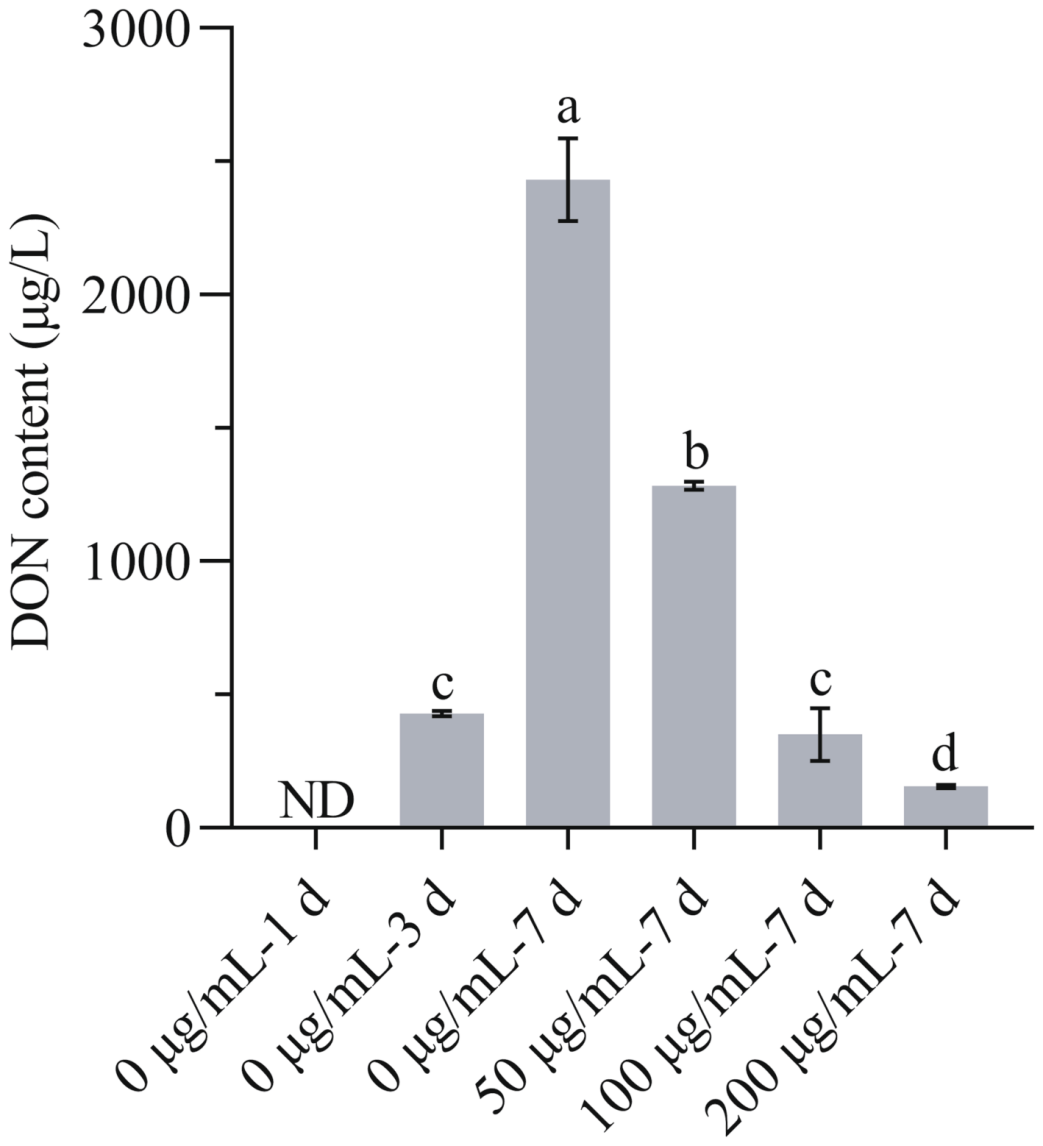
Effect of HMB on DON production of *Fusarium graminearum*. ND, not detected. a-d significant difference (*p* < 0.05) according to Duncan’s multiple range test.

The change in DON content is another aspect of the antifungal effect of HMB. As in Figure 7, at 1 day, the content of DON in the control group was not detected, and in the following week, the content increased in a time-course test. Although the DON content of the 50 μg/mL group at 7d was higher than that of 0 μg/mL-3d (428.00 ± 9.90 μg/L), the value was much lower than that of 0 μg/mL-7d (2431.75 ± 155.21 μg/L), indicating a suppression effect of DON biosynthesis induced by HMB treatment. Furthermore, the most significant inhibition effect was observed in the 200 μg/mL group, where the DON content was only 155.8 ± 6.08 μg/L, much lower than 0 μg/mL-3d. Qi et al. reported that salicylic acid at higher concentrations (≥0.5 mM) rather than lower concentrations could strongly reduce the production of DON (Qi et al., 2012). Other studies reported a dose-dependent manner of antifungal agents including z-5, a natural-like phenolic compound (Ma et al., 2022), hop essential oil (Jiang et al., 2023), thyme oil (Qi et al., 2023), myriocin (Shao et al., 2021), etc. To examine the relationship between lipid peroxidation and DON production, correlation analysis was carried out. Supplementary Table S2 shows the content of DON and MDA on 5th day after inoculation, Pearson’s correlation analysis showed the correlation coefficient value (*r*) was −0.892, indicating that the lipid peroxidation and DON production was strongly correlated. Therefore, these results suggested that HMB exerted a strong effect on DON biosynthesis, and further experiments (qRT-PCR) are required to demonstrate the oxidation and anti-toxigenic mechanism.

### Antifungal effect of HMB *in vitro* on wheat grains

**Figure 8:**
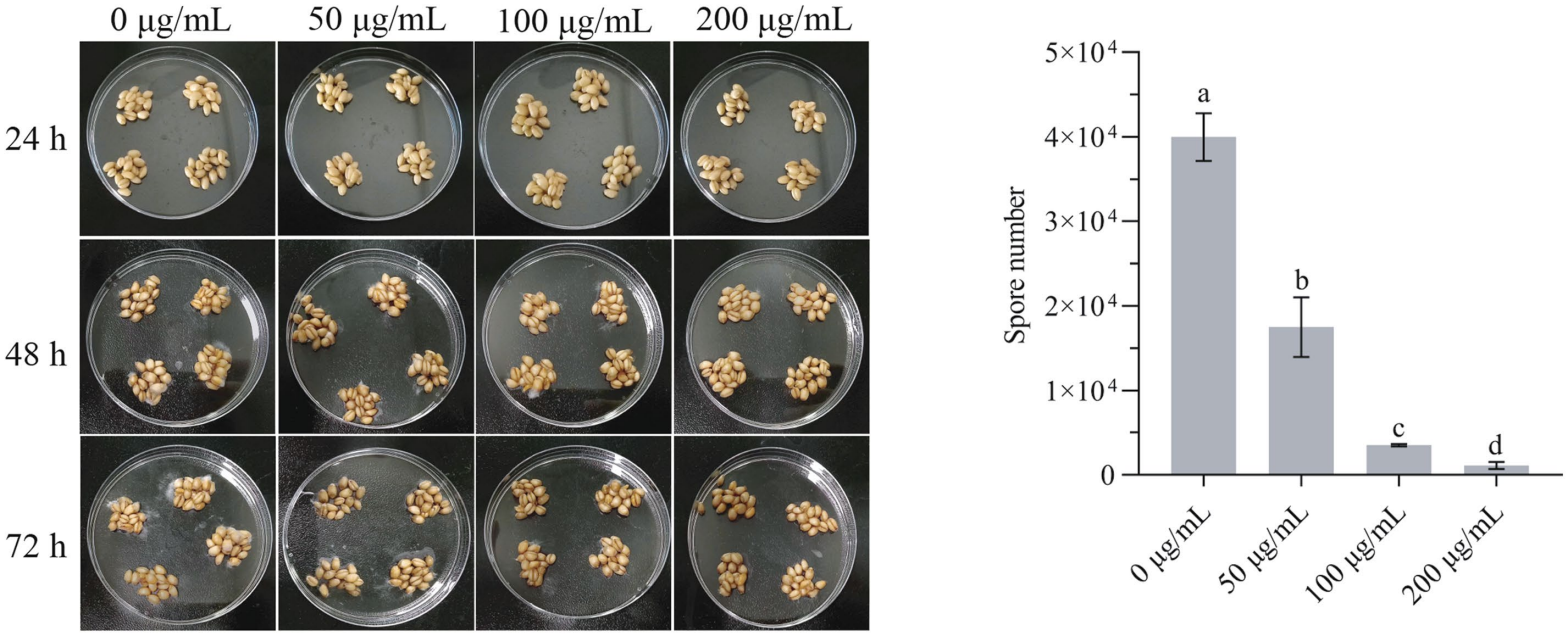
Inhibitory effect of HMB on the growth of *Fusarium graminearum* on wheat grains. a-d significant difference (*p* < 0.05) according to Duncan’s multiple range test.

Wheat is extremely susceptible to *F. graminearum* due to its high moisture content and sufficient nutrients for the growth of *F. graminearum*. As the strong inhibition effect of HMB has been proved in mycelia growth, an antifungal test on wheat grains was carried out. As in Figure 8A, obvious germination of spores started at 48 h in the control group and the 50 μg/mL treatment group, whereas no mycelia were observed in other HMB treatment groups. At 72 h, spores in all the HMB treatment groups lower than 200 μg/mL were germinated, indicating that HMB at a higher concentration is effective in restraining spore germination on wheat grains. The number of spores after 72 h cultivation was also quantified, as shown in Figure 8B. Spores in the control group were about 4 × 10^4^, HMB at 50 μg/mL decreased the number of spores by about 60%, and this inhibition was enhanced with a higher concentration of HMB, and only 1,100 spores were detected in the 200 μg/mL group. Therefore, in combination with our previous study (Li et al., 2019), HMB could effectively suppress the germination of both *A. flavus* spores on corn kernels and *F. graminearum* spores on wheat grains.

## Conclusion

To sum up, this study for the first time revealed that the inhibition of HMB against the growth of *F. graminearum* was the strongest in comparison with vanillin and *o*-vanillin. The cell membrane was a potential antifungal target of HMB, and the antifungal mechanism was involved with lipid peroxidation and oxidative stress. Meanwhile, the production of DON was significantly reduced, and the upstream molecular events of which are probably interesting to be explored in future works. In combination with the promising antifungal effect of HMB on infected grain crops, the utilization forms of HMB and the practical antifungal activity in the storage of agricultural products will be further investigated.

## Data availability statement

The original contributions presented in the study are included in the article/Supplementary material, further inquiries can be directed to the corresponding author.

## Author contributions

QL: Conceptualization, Writing – original draft, Writing – review & editing. CW: Data curation, Formal analysis, Investigation, Writing – original draft. HX: Investigation, Writing – original draft. YZ: Investigation, Writing – original draft. YX: Supervision, Writing – review & editing.
