# Supplementary material for: 2-Hydroxy-4-methoxybenzaldehyde, a more effective antifungal aroma than vanillin and its derivatives against Fusarium graminearum, destroys cell membranes, inhibits DON biosynthesis, and performs a promising antifungal effect on wheat grains
Source: Front Microbiol. 2024 Feb 26;15:1359947. doi: 10.3389/fmicb.2024.1359947 (PMC10925628; doi:10.3389/fmicb.2024.1359947)
Supplement: Supplementary file 1 [file Table_2.DOCX]

Table S1. Antifungal activity of different compounds against *F. graminearum*

| HMB concentration (μg/mL) | Mycelial growth inhibition rate (%) | | |
| --- | --- | --- | --- |
|  | 24h | 48h | 72h |
| 0 | 0.00±0.00 | 0.00±0.00 | 0.00±0.00 |
| 40 | 70.67±4.16 | 56.15±3.52 | 48.15±7.40 |
| 80 | 83.33±2.31 | 70.51±2.35 | 64.20±2.14 |
| 120 | 100.00±0.00 | 87.18±3.64 | 83.95±2.14 |
| 160 | 100.00±0.00 | 97.43±3.20 | 95.06±2.14 |
| 200 | 100.00±0.00 | 100.00±0.00 | 100.00±0.00 |
| Vanillin concentration (μg/mL) |  |  |  |
| 0 | 0.00±0.00 | 0.00±0.00 | 0.00±0.00 |
| 200 | 62.67±3.06 | 46.92±2.04 | 36.94±4.75 |
| 400 | 73.33±3.06 | 62.57±2.35 | 61.71±3.12 |
| 800 | 98.67±2.31 | 94.87±1.94 | 83.95±2.14 |
| 1200 | 100.00±0.00 | 100.00±0.00 | 95.06±2.14 |
| 1600 | 100.00±0.00 | 100.00±0.00 | 100.00±0.00 |
| *o*-Vanillin concentration (μg/mL) |  |  |  |
| 0 | 0.00±0.00 | 0.00±0.00 | 0.00±0.00 |
| 50 | 55.33±4.16 | 33.59±2.70 | 24.32±7.52 |
| 100 | 75.33±1.15 | 48.46±22.77 | 43.69±4.13 |
| 200 | 82.66±2.00 | 77.95±2.35 | 76.58±9.20 |
| 300 | 100.00±0.00 | 100.00±0.00 | 95.50±2.06 |
| 400 | 100.00±0.00 | 100.00±0.00 | 100.00±0.00 |
