# Supplementary material for: 2-Hydroxy-4-methoxybenzaldehyde, a more effective antifungal aroma than vanillin and its derivatives against Fusarium graminearum, destroys cell membranes, inhibits DON biosynthesis, and performs a promising antifungal effect on wheat grains
Source: Front Microbiol. 2024 Feb 26;15:1359947. doi: 10.3389/fmicb.2024.1359947 (PMC10925628; doi:10.3389/fmicb.2024.1359947)
Supplement: Supplementary file 2 [file Table_3.DOCX]

Table S2 Content of MDA and DON on 5^th^ day.

| HMB concentration (μg/mL) | MDA concentration (nmol/g) | DON concentration (μg/L) |
| --- | --- | --- |
| 0 | 31.85±0.56 d | 733.73±11.69 a |
| 50 | 36.71±0.86 c | 308.72±3.22 b |
| 100 | 38.37±0.38 b | 151.00±5.62 c |
| 200 | 45.76±0.84 a | 53.90±4.38 d |
| Correlation coefficient value (*r*) | -0.892 | |
